# Atypical Teratoid/Rhabdoid Tumors in Adults: A Case Report and Treatment-Focused Review

**DOI:** 10.4021/jocmr535w

**Published:** 2011-04-04

**Authors:** Nicole A. Shonka, Terri S. Armstrong, Sujit S. Prabhu, Amanda Childress, Shauna Choi, Lauren A. Langford, Mark R. Gilbert

**Affiliations:** a Division of Oncology and Hematology, University of Nebraska Medical Center, 987680 Nebraska Medical Center, Omaha NE 68198-7680, USA; b Department of Neuro-Oncology, University of Texas at MD Anderson Cancer Center, 1400 Holcombe, Unit 431, Houston, Texas 77030, USA; c Department of Neurosurgery, University of Texas MD Anderson Cancer Center, 1400 Holcombe, Unit 442, Houston, Texas 77030, USA; d Brain and Spine Center, University of Texas at MD Anderson Cancer Center, 1515 Holcombe Blvd, Houston, TX 77030, USA; e Division of Pharmacy, Department of Neuro-Oncology, University of Texas at MD Anderson Cancer Center, 1400 Holcombe, Unit 431, Houston, Texas 77030, USA; f Department of Pathology/Neuropathology, University of Texas at MD Anderson Cancer Center, 1400 Holcombe, Houston, Texas 77030, USA

## Abstract

Atypical teratoid/rhabdoid tumor is predominantly a childhood tumor and has only been rarely reported in adults; therefore, treatment regimens are often extrapolated from the pediatric experience. Typically, children are treated with craniospinal radiation therapy which is often followed by systemic chemotherapy. Employing pediatric regimens to treat this tumor in adult patients poses a particular risk for myelosuppression, as the prescribed doses in pediatric protocols exceed those tolerated by adults, and conventional craniospinal radiation can be associated with prolonged myelotoxicity and a depletion of the bone marrow reserve in vertebrae of adults. Here we present a case of a woman with a pineal region atypical teratoid/rhabdoid tumor, an unusual adult cancer presenting in an atypical location. This is followed by a review of the disease in adult patients with an emphasis on treatment and suggestions to minimize myelotoxicity.

**Keywords:**

Atypical rhabdoid tumor; AT/RT; Pineal tumor; Adult

## Case Report

**Figure 1.**
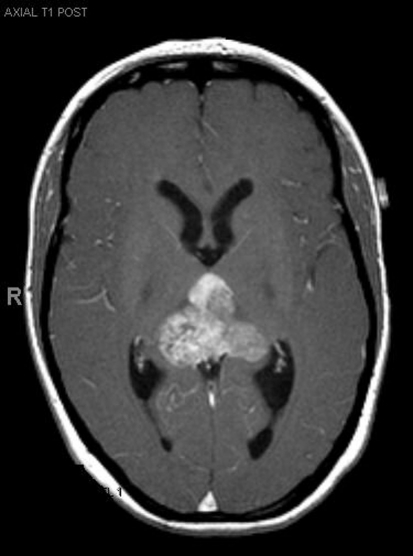
Pre-operative MRI.

**Figure 2.**
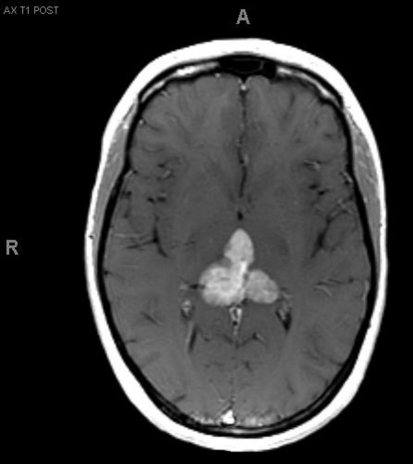
MRI after first resection.

**Figure 3.**
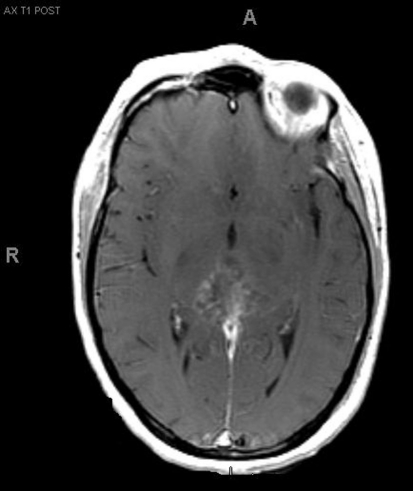
MRI after second resection.

A 33-year-old right-handed woman developed the sensation of fullness in her head followed months later by blurred vision that progressed to double vision. Brain MRI identified a large pineal mass and hydrocephalus (Fig. 1). A subtotal resection of the mass with concurrent placement of a ventriculoperitoneal shunt was performed at an outside institution (Fig. 2). Pathology suggested an epithelioid neoplasm, but a definitive diagnosis could not be made. Three weeks later, the tumor had regrown to its original size and a repeat supracerebellar infratentorial craniotomy was performed at MD Anderson Cancer Center with a near complete resection of the pineal mass (Fig. 3). Immunohistochemistry was positive for epithelial membrane antigen and smooth muscle actin. An antibody against the hSNF5/INI1 protein was negative in tumor cell nuclei. These findings confirmed the diagnosis of atypical teratoid/rhabdoid tumor (AT/RT), WHO grade IV. Cerebrospinal fluid and spinal MRIs were negative for tumor dissemination.

Peripheral blood stem collection was performed prior to the initiation of chemotherapy. The patient underwent craniospinal radiation therapy (CSRT) and post-radiation MRI showed a modest decrease in the size of the residual tumor. Chemotherapy with Ifosphamide, Etoposide and Carboplatin (ICE) was given in 3-week cycles. After two cycles, brain MRI showed a partial tumor response. After her eighth cycle there was evidence of tumor progression with recurrence in the thalamus as well as along the occipital horn of the lateral ventricle. The treatment regimen was changed to doxorubicin, vincristine and temozolomide. The patient continues on this treatment regimen, and remains clinically and radiographically stable 18 months after the initial diagnosis.

## Review of the Literature

### The history of AT/RT

Beckwith and Palmer, in 1978, first coined the term ‘rhabdoid tumor’ to describe a histological variant of Wilm’s tumor found primarily in infants that was associated with an extremely poor prognosis [1]. The name was derived from its similarity in gross tumor appearance to a rhabdomyosarcoma; however, the cells differed from the expected morphological and immunohistochemical features of muscle [2]. A tumor composed of rhabdoid cells in the central nervous system (CNS), was first reported in 1985 [3]. The name ‘atypical teratoid/rhabdoid tumor’ (AT/RT) exemplifies the tumors disparate mixtures of rhabdoid, primitive neuroepithelial, mesenchymal and epithelial components [4]. AT/RT is much more frequently seen in infants and young children than older children and is rare in adults. AT/RT has an overall incidence of 1 - 2% of all brain tumors in children [4, 5]. They are estimated to account for over 10% of CNS tumors in infants, with a male preponderance up to the age of 3 which then seems to disappear [6, 7]. There exists a rhabdoid tumor predisposition syndrome which can be inherited in an autosomal dominant fashion, but most commonly occurs sporadically. The genetic form of AT/RT results from a germline loss of function mutations in INI1, also known as SMARCB1, a tumor suppressor gene at 22q11.23 [8]. This syndrome commonly manifests in tumors of the kidneys, brain and soft tissues. There have been just over 30 adult cases reported in the literature to date [9-31] (summarized in Table 1). Clinical presentation varies with tumor location in adults where a variety of primary locations have been reported.

**Table 1:** Adult Patients With AT/RT in the CNS

Author (year) (ref)	Age (yr)/Sex	Tumor Location	Immunostains	LMD	Diagnosis Confirmed	INI1 Analysis	Primary Treatment	Secondary Treatment	TTP (mos)	OS (mos)
Balaton (1987) [27]	59M	Paravertebral	CK Vim	Y	IHC	N	None	n/a	n/a	0.5
Horn (1992) [9]	21M	L temporal	EMA Vim	N	IHC	N	STR	STR CT	48	72
Cossu (1993) [26]	18M	L frontal	CK EMA Vim	N	IHC	N	GTR CT	STR	5	18
Fisher (1996) [10]	32M	L caudate	cGFAP S100 Vim	Y	IHC	N	None	n/a	n/a	1
Ashraf (1997) [11]	34M	L parietal	Vim	N	IHC	N	STR RT	STR	2	6
Byram (1999) [12]	35M	L temporal	NA	N	NA	NA	GTR RT	GTR	36	60
Sugita (1999) [34]	27M	Pineal region	chrA EMA NSE S100 Vim	N	IHC	N	STR CRT ACNU	STR	18	24
Kuge (2000) [13]	32F	Suprasellar	CK EMA SMA Vim	Y, r	IHC	N	STR	CSRT CDDP, VP16, IFN IT mtx	1	8+
Arrazola (2000) [14]	20M	L parietal	CK EMA S100 Vim	N	IHC	N	GTR	GTR CSRT	3	84
Lutterbach (2001) [15]	30F	Cerebellum	CK S100 Vim	N	IHC	N	GTR RT	SRS Tmz	6	11
Bruch (2001) [16]	21F	Spinal cord	CK EMA Vim	N	IHC, 22qdel	N	NA	NA	NA	6
Bruch (2001) [16]	34F	Parietal	CK EMA Vim	N	IHC, 22qdel	N	NA	NA	NA	6
Pimentel (2003) [17]	31F	R parietal	EMA GFAP S100 Vim alpha1ac/at	Y	IHC	N	STR	CSRT ICE	1	6
Kachhara (2003) [19]	35M	Thalamus	Vim	N	IHC	N	STR RT	-	2	NA
Kawaguchi (2004) [18]	22M	L cerebellum	CK EMA NSE SMA Vim	Y	IHC	N	STR CSRT ICE IT mtx	-	n/a	24+
Raisanen (2005) [20]	45M	R cerebellum	CK EMA S100 SMA Vim	N	IHC	Y	Sx CT RT	NA	NA	15+
Raisanen (2005) [20]	20F	Sella	CK EMA SMA Vim	N	IHC	Y	Sx R CT	CT	NA	28+
Raisanen (2005) [20]	31F	Sella	CK EMA Vim	N	IHC	Y	Sx RT	NA	NA	9
Erickson (2005) [21]	20F	R occiput	EMA GFAP SMA Vim	N	IHC	Y	GTR	RT	NA	NA
Chen (2006) [28]	19M	Post fossa	NA	NA	NA	NA	GTR	CSRT	7	56.5
Ingold (2006) [22]	45F	Pineal	CK EMA SMA Vim	Y, r	IHC	Y	GTR CRT	GTR	6	7
Rezanko (2006) [23]	27M	R frontal	EMA S100 Vim	Y, r	IHC	N	GTR RT	-	4	4
Chacko (2007) [29]	23M	R frontal	EMA SMA Vim	N	IHC	Y	STR RT	GTR	1	2
Zarovnaya (2007) [24]	43F	Spinal cord	EMA	Y, r	IHC	Y	STR RT	CSRT Tmz IFN	2	30
Makuria (2008) [30]	23M	L temporal	CK EMA NF SMA Syn Vim	N	IHC	Y	Sx RT CT	-	n/a	30+
Makuria (2008) [30]	25F	R frontal	NF SMA Syn Vim	N	IHC	Y	GTR	GTRx5 GKS RT	24	204+
Makuria (2008) [30]	42M	R frontalparietal	CK EMA Vim	N	IHC	Y	STR RT CT	-	-	18+
Makuria (2008) [30]	37M	R frontalparietal	Vim	N	IHC	Y	NA	NA		NA
Arita (2008) [31]	56F	Sella/cav sinus	EMA NF Vim	Y, r	IHC	Y	STR SRS	CSRT	12	23
Samaras (2009) [25]	18M	R frontaltemporal	EMA GFAP SMA Vim	N	IHC	Y	GTR RT	-	-	4
Our patient	33F	Pineal region	EMA SMA	N	IHC	Y	GTR CSRT ICE	Tmz + Vcr	8	18+

LMD: leptomeningeal disease; Y: yes; N: no; Y, r: yes at recurrence; IHC: Immunohistochemistry; NA: not available; STR: subtotal resection; GTR: gross total resection; CT: chemotherapy; CRT: chemoradiation; RT: radiation therapy; CSRT: craniospinal radiation therapy; IT: intrathecal; Sx: surgery (extent unknown); n/a: not applicable; CK: cytokeratin; Vim: vimentin; EMA: epithelial membrane antigen; cGFAP: cytoplasmic glial fi brillary acid protein; SMA: smooth muscle actin; chrA: chromogranin A; NSE: neuron-specifi c enolase; alpha1ac/at: alpha-1 antichymotrypsin/antitrypsin; NF: neurofi bromin; ACNU: Nimustine; ICE: ifosphamide, carboplatin, etoposide; mtx: methotrexate; SRS: stereotactic radiosurgery; CDDP: Cisplatin; VP16: etoposide; IFN: interferon-gamma; Tmz: temozolomide; GKS: gamma-knife surgery; Vcr: vincristine.

No data exists to support imaging characteristics that differentiate AT/RTs from other primitive neuroectodermal tumors [32]. Report from the AT/RT workshop in 2002 noted that half of all AT/RTs are in the posterior fossa, although the tumor has been noted throughout the nervous system and in extramedullary sites. Tumors can be extraaxial and invade adjacent structures such as the meninges as well [32]. In adults, these are primarily found in the cerebral hemispheres [33] and are rare in the cerebellum and spinal cord [16, 17, 19, 21, 25]. Similar to our case, two other adult AT/RTs have been found in the pineal region [23, 34].

Computed tomography (CT) usually shows a hyperdense mass that intensely enhances after administration of intravenous contrast. On T1-weighted magnetic resonance imaging (MRI) the mass is commonly isointense with hyperintense areas that result from intratumoral bleeding. The T2 imaging is more heterogenous with hypointense to hyperintense areas indicating a mixture of necrosis, hemorrhage, cystic changes, and calcifications [32, 35]. Peritumoral edema was variable in the meta-analysis of 133 patients done by Oka [36]. MR spectroscopy shows a marked elevation of choline and low or absent N-acetylaspartate (NAA) and creatinine, as would be expected. In a review of thirteen patients ages 4 months to 15 years with AT/RT, all tumors except one enhanced with contrast on MRI [35].

AT/RTs consist of a combination of rhabdoid, primitive neuroepithelial, mesencymal and epithelial cells. Approximately one-third contain epithelial or mesenchymal cells, and only 10% are comprised purely of rhabdoid cells [32]. This heterogeneity makes the discrimination between AT/RT and the other tumors of embryonal tissue, namely medulloblastoma and primitive neuroectodermal tumor (PNET), difficult using histologic criteria [37, 38]. Thus far, the histogenesis of this tumor has remained elusive [10, 32, 39]. Proliferative activity is high, and Ki-67/MIB-1 labelling indices averaged 63.9% in a series of pediatric patients [40], and have ranged from less than 20% to 80% [9, 12, 16, 34].

Immunohistochemistry (IHC) is positive for rhabdoid cell markers including epithelial membrane antigen (EMA), vimentin, and smooth-muscle actin (SMA) in the majority of tumors and markers for germ-cell tumors such as alpha-fetoprotein and placental alkaline phosphatase are consistently negative [4, 32]. The tumors may also express glial fibrillary acidic protein, keratin, synaptophysin, and neurofilament protein [32]. IHC staining for the INI1 protein, a component of a SWI/SNF ATP-dependent chromatin-remodeling complex has been shown to be highly sensitive and specific for AT/RTs [41-43]. Versteege suggested that any loss-of-function mutations of INI1 contribute to oncogenesis after noting bi-allelic alterations of INI1 (i.e., any truncating mutation of one allele caused loss of the other allele). Monosomy 22 or deletions of chromosome band 22q11 are found in most AT/RTs, however, alterations of chromosome 22 are shared in other CNS tumors as well. PNETs may have deletions of chromosome 22 but can sometimes be differentiated from AT/RTs by the presence of chromosome 17 abnormalities [4, 42, 44].

### Prognosis

Tekautz and colleagues reported the outcomes from a series of 37 pediatric patients [7]. Event-free survival (EFS) and overall survival (OS) at two years for children aged three years or older was 78% and 89% vs 11% and 17 % for younger children. Oka performed a meta-analysis of 133 patients and found that 98 (74%) patients had succumbed to their disease within 24 months of diagnosis, with a mean OS of 8.5 months. Seventy-five percent of these patients were younger than three years of age [36]. Recently, the modified IRS-III regimen evaluated by Chi and colleagues increased two-year survival to 70% ± 10% compared to historic median survival of only 6 - 11 months [32, 39, 45]. Of the 31 adult patients whose survival data were reported in the literature, the median survival was 15 - 18 months, although it ranged widely from two weeks to over 17 years (Table 1).

### Treatment

The impact of the extent of surgical resection on outcome has not been fully studied. Packer reported from the pediatric AT/RT registry an OS of 8.5 months that lengthened to 13 months in patients who had gross total resection (GTR) of their tumors. In this report, a personal communication with J. Hilden M.D. is cited that of the eight patients in the registry with an OS of greater than 18 months, six of those had undergone a GTR [32]. Hilden’s report favors more aggressive resection as well with an OS of 20 months vs 15.25 months [46].

**Table 2:** Chemotherapy Used for AT/RT in Children

Author (year) (ref)	Age range at dx	n	First line Chemotherapy	Outcomes
Biegel (1990) [44]	6 mos - 12 mos	3	Cy, VP-16, Cis, Vcr/Cis, Cy, Vcr, Mtx, CCNU/Vcr, BCNU, Proc, Hydroxy, Cis, Ara-C	DOD 3 - 5 mos
Agranovich (1992) [54]	33 mos	1	Cis, Doxo	DOD 8 mos
Weinblatt (1992) [55]	-	1	Vcr, Act-D, Doxo, IT Mtx, IT Ara-C, IT HC	NED 5 + yrs
Hanna (1993) [56]	8 mos - 6 yrs	3	Vcr, VP-16, Cis, Cy, Vcr, nitrogen mustard, IT Mtx/Ifos, VP-16, Carbo	DOD 6 - 15 mos
Olson (1995) [47]	18 mos - 5 yrs	3	IRS III based (Vcr, Cis, Doxo, Cy, VP-16, Act-D, IT Mtx, IT Ara-C, IT HC)	NED 6 - 42 mos
Dang (2003) [57]	4 wks - 25 mos	3	Vcr, Cy, Act-D, Doxo, Vcr, VP-16, Cis, Cy, Doxo, Ifos, IT thio/VP-16, Cis	DOD 5 mos - 1 year
Izychka-Swieszewska (2003) [58]	5 mos		8-in-1 (Vcr, CCNU, Proc, Hydroxy, Cis, Ara-C, Cy, MP)	DOD 8 mos
Wharton (2003) [59]	31 mos - 14 yrs	3	UKCCSG SIOP PNET (Vcr, VP-16, Carbo, Cy)/UKCCSG infant brain tumor protocol (Vcr, Carbo, Cy, Mtx, Cis)	DOD 11 mos AWD at 1 year NED 4 yrs
Zimmerman (2005) [60]	14 mos - 11 yrs	4	DFCI/IRS III modified* (Vcr, Cis, Doxo, Dexraz, Cy, VP-16, Actino, Tmz, IT Mtx, IT Ara-C, IT HC)	NED 33 mos - 4 yrs
Chen (2006) [28]	24 mos - 11.2 yrs	11	VIP (Vinb, Ifos, Cis), IT Mtx +/-, ACNU/ICE + CECAT (Cy, VP-16, Carbo, Thio)	DOD 7 - 24 mos AWD 15 - 17 mos NED 35 - 105 mos
Fidani (2009) [61]	16 mos - 8.6 yrs	8	ICE + CECAT (Cy, VP-16, Carbo, Thio)/ICE/ICE+TMZ	DOD 8 - 13 mos AWD 5 - 38 mos NED 101 - 105 mos
Gardner (2008) [62]	4 - 52 mos	13	HSI (Induction: Cis, VP-16, Cy, Vcr, Consolid: Carbo, Thio, VP-16, ASCR)/HS II (same as HS I plus Mtx in Induction)	DOD/DOC 0.5 - 11.5 mos NED 42 - 67 mos
Lassaletta (2009) [63]	8 mos		ACNS 0121 (Vcr, Cy, Carbo, VP-16) and IT depot Ara-C	DOD 2 mos
Biswas (2009) [64]	6 years		GTR, CSRT, VAC (Vcr, Doxo, Cy)	NED 24 mos
	6 years		STR, VAC	DOD < 2 mos
	5 years		STR, CSRT, VAC	AWD?
	18 mos		STR, ICE	DOD < 2 mos
Chi (2009) [45]	4 mos - 8.4 yrs	18	GTR, CRT, modified IRS-III (Vcr, Dactino, Cy, Cis, Doxo, Tmz, IT Mtx, Ara-C, HC)	DOD/DOC 1 - 24 mos AWD 17 - 34 mos NED 18 - 40 mos
Ertan (2009) [65]	8 mos - 8 yrs	2	ICE	DOD 4 - 5 mos
Wang (2009) [66]	16 - 42 mos		Cis, Ifos, VP-16, IT ACNU/TMZ**	AWD 12 mos NED 59 mos

Ara-C: cytarabine; AWD: alive with disease; Bx: biopsy; Carbo: carboplatin; Cis: cisplatin; CSRT: craniospinal radiation therapy; Cy: cyclophosphamide; DOC: dead of complications; DOD: dead of disease; EBRT: external beam radiation therapy; GTR: gross total resection; HC: hydrocortisione; Hydroxy: hydroxyurea; ICE: ifosphamide, carboplatin, etoposide; Ifos: ifosphamide; IT: intrathecal; Mtx: methotrexate; NED: no evidence of disease; Proc: procarbazine; STR: subtotal resection; Tmz: temozolomide; Vcr: vincristine; VP-16: etoposide. * One patient received this second-line. ** One patient only received second-line chemotherapy.

Treatment paradigms for adult patients have been extracted from the pediatric literature. Chemotherapeutic regimens used in the pediatric population vary, but regimens commonly utilize vincristine with an alkylating and a platinum agent. Table 2 highlights several regimens and outcomes in pediatric patients.

Excluding our patient, there have been 31 adult cases reported in the (English) literature. There is no information available regarding treatment given to three of these patients, and no survival information was provided for three patients. Of the 28 adult patients in whom treatment was reported, 14 (50%) received chemotherapy, either concurrent with or after radiation therapy. Temozolomide and ICE were commonly used. Survival in patients who received chemotherapy ranged from 6 months to 17 years, with a median survival of 24 months. Those who received surgery and radiation therapy without chemotherapy had a survival between 2 and 7 years, with a median survival of 9 months. In a small case series, we cannot confirm the superiority of one regimen or even a benefit from chemotherapy. The data supports the importance of preserving bone marrow function so that systemic chemotherapy remains a viable option.

Much of the data regarding treatment has focused on chemotherapy, since the majority of patients diagnosed with AT/RT are under two to three years old when RT is avoided if possible. Those patients over the age of three are routinely given RT, often in the form of CSRT as leptomeningeal disease (LMD) is often present at diagnosis and is common at recurrence. The AT/RT registry shows a high rate of local recurrence, and those who survived more than 18 months were more likely (75%) to have received RT [32]. Patients are given varying doses between 40 - 60 Gy, and stereotactic radiosurgery has been used for recurrent disease when resection is not feasible [47]. There are no data on the response to RT in adult AT/RT but of 13 patients in a case series from the Childrens Hospital of Philadelphia, only two patients had an objective response [4].

In the report on 42 pediatric cases by Hilden, 13 patients underwent stem cell rescue as part of their primary treatment, which underscores the significant myelosuppression of treatment regimens for AT/RT [46]. Approximately 40% of adult bone marrow resides in the spine [48]. In our patient, we were concerned about the additive myelotoxicity of CSRT and chemotherapy and so a stem cell harvest was performed and the patient was given radiation with protons. Unlike conventional photon radiotherapy, proton radiotherapy focuses the maximum dose to the target tissue while sparing normal tissues from much of the entry dose and the entire exit dose. This occurs as protons lose only a small amount of their energy in tissue until they reach the target tissue, after which the residual energy is rapidly lost, resulting in a steep treatment gradient [49]. Therefore, the use of proton-beam radiation for craniospinal treatment may allow a partial sparing of vertebral body radiation exposure, lessening the impact on this major component of hematopoeisis and lessening the degree of myelotoxcity.

Laboratory studies have focused on establishing an AT/RT in vitro cell culture model on which preclinical studies can investigate both chemotherapeutic agents as well as targeted therapies [50-53]. Insulin-growth factor-1 receptor (IGF-1R) inhibition has been shown to sensitize cells to both chemotherapy and radiation [53]. Using AT/RT cells cultured from CSF, Narendran noted growth inhibition with low concentrations of arsenic trioxide, Prima-1 (targets mutant p53 proteins), oxaliplatin, cisplatin and rebeccamycin. Thalidomide, etoposide, cytarabine and paclitaxel had intermediate MICs. The optimal treatment remains to be defined, but the increasing recognition of this disease and the development of good laboratory models will hopefully accelerate therapeutic advances.

### Conclusion

AT/RT remains a rare adult disease. However, as our knowledge of AT/RTs increases we anticipate that there will be more standardization of treatment. Aggressive resection followed by multimodality treatment appears to yield more long-term survivors. In adults, although the use of RT does not convey the same devastating developmental arrest, there are still reasons to minimize the effects to normal tissues particularly bone marrow, supporting the use of proton radiation, particularly since CSRT is a standard treatment. Although the optimal chemotherapy regimen has not been defined for adults with AT/RT, several regimens have been used with evidence of activity. Further advances in treatment will likely require more laboratory studies generating novel treatment regimens for clinical trial testing. The rarity of adult AT/RT suggests that treatment regimens will continue to rely on advances in pediatric treatments.

## Conflicts of Interest

The authors have no relevant conflicts of interest to disclose.
